# Src is the primary target of aripiprazole, an atypical antipsychotic drug, in its anti-tumor action

**DOI:** 10.18632/oncotarget.23192

**Published:** 2017-12-08

**Authors:** Mi Seon Kim, Byong Chul Yoo, Woo Seok Yang, Sang Yun Han, Deok Jeong, Jun Min Song, Kyung Hee Kim, Adithan Aravinthan, Ji Hye Kim, Jong-Hoon Kim, Seung Cheol Kim, Jae Youl Cho

**Affiliations:** ^1^ Department of Genetic Engineering, Sungkyunkwan University, Suwon 16419, Republic of Korea; ^2^ Colorectal Cancer Branch, Research Institute, National Cancer Center, Goyang 10408, Republic of Korea; ^3^ School of Medicine, Keimyung University, Daegu 42601, Republic of Korea; ^4^ Department of Physiology, College of Veterinary Medicine, Chonbuk National University, Iksan 54596, Republic of Korea; ^5^ Department of Obstetrics and Gynecology, Ewha Womans University Mokdong Hospital, Ewha Womans University School of Medicine, Seoul 07985, Republic of Korea

**Keywords:** aripiprazole, antipsychotic drug, antitumor activity, apoptosis, Src

## Abstract

Aripiprazole (ARP) is an atypical anti-psychotic drug widely used to treat schizophrenia and bipolar disorder. The pharmacological effects of ARP on cancer cells are still poorly understood. In this study, anti-cancer effects of ARP on various malignant tumor cells and its molecular mechanism were further carefully examined by using cell proliferation assay, xenograft mouse model, immunoblotting analysis, migration assay, luciferase reporter gene assay, kinase assay, and overexpression strategy. Treatment with ARP induced cytotoxicity in U251 glioma cells, MKN-1 gastric adenosquamous carcinoma cells, and CT26 colon carcinoma cells. ARP suppressed cell proliferation of LN428, MDA-MB-231, and HEK293 cells. Pro-apoptotic factors active caspase-3, -8, and -9, as well as p53, were upregulated, whereas the protein and mRNA levels of anti-apoptotic factor B-cell lymphoma 2 (Bcl-2) decreased. In agreement with the *in vitro* results, ARP compound also significantly suppressed the growth of tumor masses formed by injecting CT26 colon cancer cells into mice. ARP treatment also effectively decreased the migratory ability of U251 glioma cells by downregulating metalloproteinase-9. Levels of phosphorylated Src, phosphorylated phosphatidylinositide 3-kinase (PI3K), and phosphorylated signal transducer and activator of transcription 3 (STAT3) were significantly decreased following ARP treatment. ARP compound reduced the kinase activity of Src. Our studies suggest that Src may be an important target molecule linked to the antitumor effects of ARP.

## INTRODUCTION

Src is one of several oncogenic tyrosine kinases. It contains SH2 and SH3 domains that are important for its activation [1]. Src is activated in many types of cancer to influence survival and metastasis [2, 3, 4]. As a result, Src inhibition has been explored for the development of anticancer drugs [5, 6]. In a previous study, we verified that adenosine dialdehyde, an inhibitor of transmethylation-suppressive adenosylhomocysteine hydrolase, suppresses tumorigenesis by cross-regulation of the actin cytoskeleton and Src kinase [7]. We also reported that four synthetic compounds derived from *Cordyceps bassiana* induce apoptosis in cancer cell lines through regulation of Src [8, 9, 10, 11].

**Figure 1:**
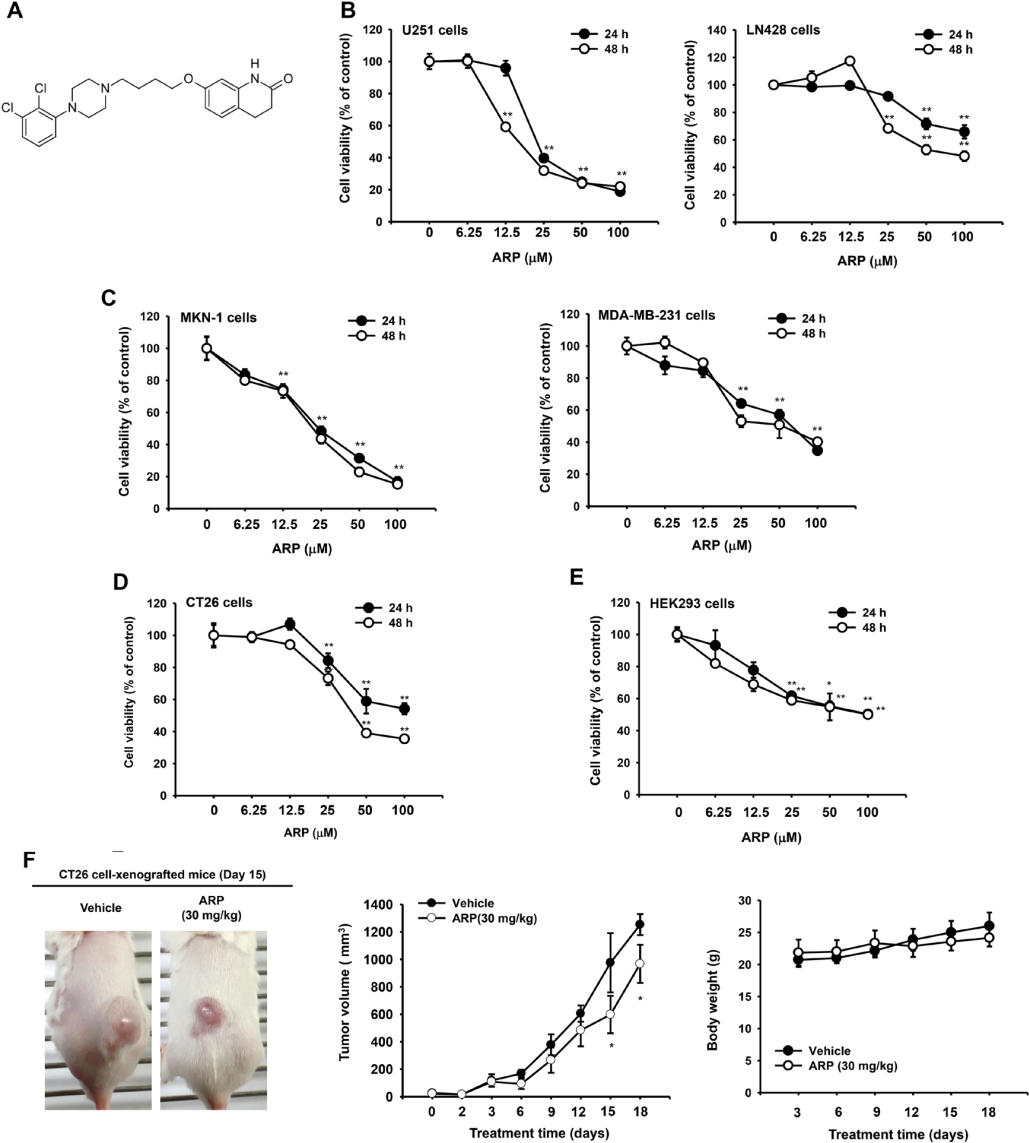
*In vitro* and *in vivo* anti-cancer effects of aripiprazole (ARP) (**A**) Chemical structure of ARP, an antipsychotic to treat schizophrenia and bipolar disorder. (B, C, D, and E) Cytotoxic effect of ARP evaluated by conventional MTT assays. Viability of glioma U251 (**B** left) and LN428 (B right) cell lines, human gastric cancer cell line MKN-1 (**C** left), breast cancer line MDA-MB-231 (C right), mouse CT21 colon cancer cell line (**D**), and noncancerous HEK293 (**E**) cell line following ARP treatment for 24 and 48 h. (**F**) *In vivo* ARP anti-cancer activity evaluated using xenograft mice bearing CT26 cell-derived cancers. Mice were subcutaneously injected with 10,000 CT26 cells in 0.1 ml. Photograph of mice with CT26 cell-derived cancers by digital camera (upper). Tumor volumes were determined using digital calipers every 1, 2, or 3 days for 18 days (middle). Body weight was measured for 18 days at 3-day intervals (lower). ^*^P < 0.05 and ^**^P <0.01 compared with normal group. Data are presented as the mean ± standard error of the mean (SEM) of three independent experiments conducted in triplicate. ^*^:*p* < 0.05 and ^**^:*p* < 0.01 compared to normal or control groups.

Aripiprazole (ARP) (Figure 1A) is a synthetic drug developed by Otsuka Pharmaceuticals (Tokyo, Japan) [12]. An atypical antipsychotic, ARP is used to treat psychotic disorders such as schizophrenia, psychotic episodes related to depression, bipolar disorder, and delayed sleep phase syndrome [13]. ARP regulates the monoaminergic system by stabilizing dopamine D2 and 5-hydroxytryptamine as a partial dopamine agonist [14]. ARP also acts as an anti-oxidative drug and a gastroprotective agent [15, 16, 17, 18]. However, the pharmacological effects of ARP in cancer are poorly understood. Therefore, we determined the effects of ARP treatment on the proliferation of various cancer cell types including glioma cells and aimed to understand the molecular mechanism of its anti-cancer activity.

## RESULTS

### ARP induces cytotoxicity in cancer cell lines and inhibits *in vivo* tumor growth

ARP reduced the viability of the glioma cell lines U251 and LN428, the gastric cancer cell line MKN-1, the breast cancer cell line MDA-MB-231, the colon carcinoma cell line CT26, and the human embryonic kidney cell line HEK293 cells. Effects were dose and time dependent (Figure 1B, 1C, 1D, and 1E). Depending on treatment time (24 to 48 h), IC_50_ values for ARP were 17.7 µM to 80.6 µM (Table 1). Among cell lines tested, the glioma cell line U251 cells were the most sensitive to ARP treatment (Figure 1B, left panel). LN428 cells were relatively resistant to ARP at 24 h, with IC_50_ values greater than 100 µM (Table 1). Since CT26 cells with IC_50_ values of 40 to 56 µM (Table 1) are N-nitroso-N-methylurethane-induced, undifferentiated mouse colon carcinoma cells [19], a mouse model was established using the cells and it was revealed to exhibit an increase in tumor volumes and animal body weight (Figure 1F), as reported previously [20]. In fact, orally administered ARP (30 mg/kg) significantly reduced the enhanced tumor volume (Figure 1F, middle panel), without suppression of body weight (Figure 1F, lower panel).

**Table 1:** Inhibitory activity (IC_50_ values) of ARP on cancer cell proliferation

Cell line	IC_50_ values (µM)	
	24 h	48 h
U251 cells	22.7	17.7
LN428 cells	> 100	80.0
HEK 293 cells	> 100	> 100
CT26 cells	56.0	40.4
MKN-1 cells	24.2	22.3
MDA-MB-231 cells	82.3	80.6

### ARP induces apoptosis in U251 glioma cells

**Figure 2:**
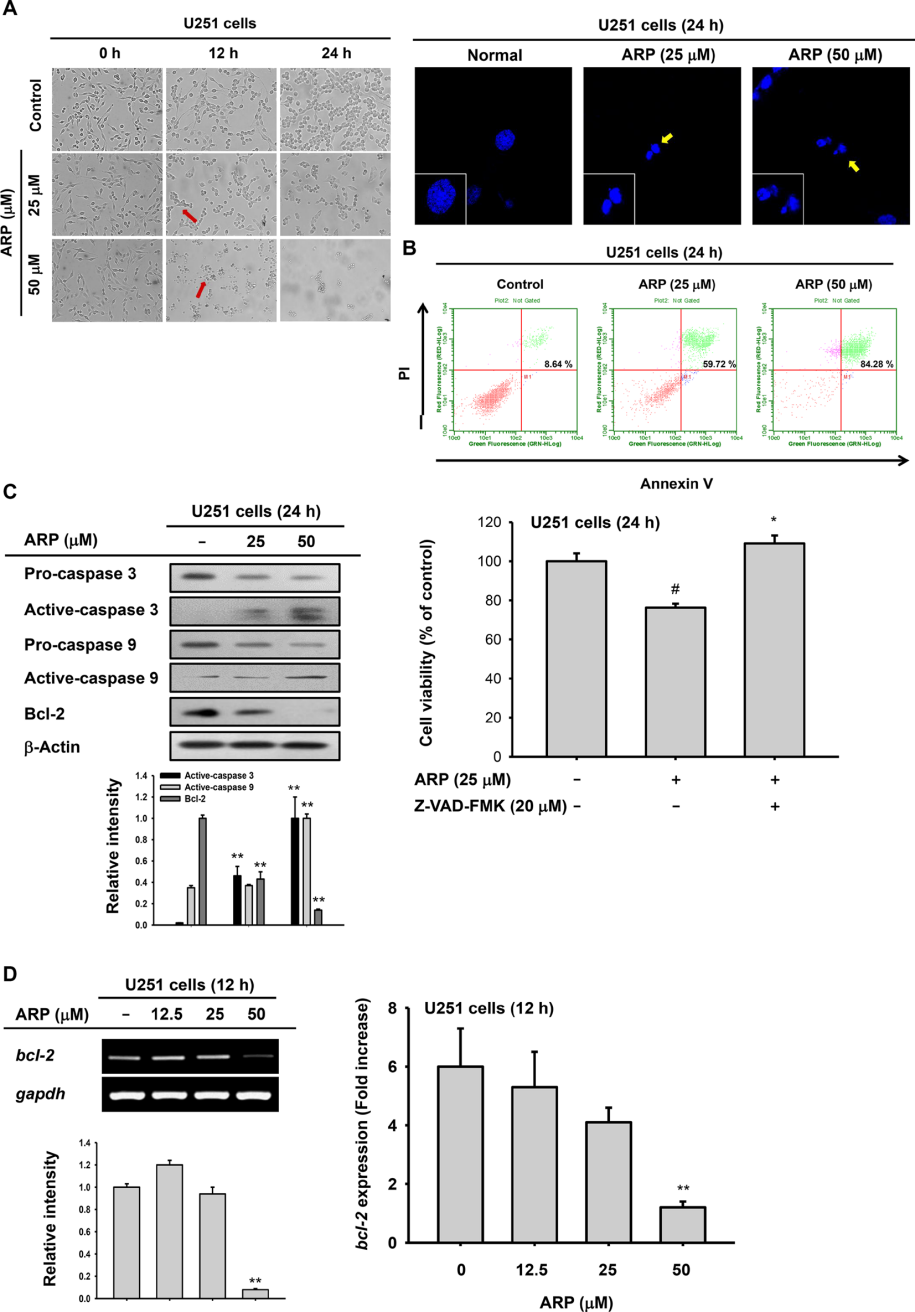
Pro-apoptotic effect of aripiprazole (ARP) in U251 cells (**A**) Morphological changes from apoptosis in ARP-treated U251 glioma cells (1 × 10^5^ cells/ml) incubated for 0, 12, and 24 h, visualized by microscopy (left). U251 glioma cells were treated with ARP for 24 h and stained with Hoechst dye. Nuclei were detected by confocal microscopy (right). (**B**) Fluorescein isothiocyanate-annexin V and propidium iodide (PI) staining confirmed ARP-induced apoptosis in U251 glioma cells. Percentage of late-apoptosis (annexin V-PI double-positive) cells by flow cytometer. (**C** left panel) Expression of apoptosis-related proteins in U251 glioma cells following ARP for 24 h, determined by immunoblots. (C right panel) Recovery activity of caspase inhibitor (Z-VAD-FMK) on ARP-mediated cytotoxicity was evaluated by conventional MTT assay. Z-VAD-FMK was pretreated 1 h before ARP treatment in U251 cells for 24 h. (**D**) The mRNA level of *bcl-2* was measured by semi-quantitative RT-PCR (left) and real time-PCR (right). Relative intensity (C and D left) was calculated using total active-caspase-3, -9, and Bcl-2 and β-actin by DNR Bio-Imaging system. ^**^P < 0.01 compared with the normal group.

To determine whether ARP-dependent cytotoxicity was due to apoptosis, several apoptotic markers were observed in ARP-sensitive U251 glioma cells. Apoptotic bodies (Figure 2A, left panel) and induced nuclear condensation (Figure 2A, right panel) were identified in U251 cells a 12 and 24 h following ARP treatment. ARP-dependent apoptosis was also monitored by annexin V and PI staining. ARP treatment dose-dependently increased the percentage of annexin V-positive and PI-positive late-apoptotic cells from 8.64% to 84.28% (Figure 2B).

ARP-dependent apoptosis was confirmed by detection of active caspase-3 and -9 in U251 cell lysates (Figure 2C). Active caspase-3 and -9 were increased in ARP-treated U251 glioma cells in a dose-dependent manner (Figure 2C left panel). Moreover, an inhibitor (Z-VAD-FMK) of caspases completely abrogated the cytotoxicity (24%) of ARP in U251 cells, as assessed by a MTT assay (Figure 2C right panel). In addition, anti-apoptotic protein Bcl-2 was reduced by ARP treatment (25 and 50 µM). We also evaluated mRNA expression of *bcl-2* under the same conditions. This gene was also downregulated in U251 cells by ARP treatment (50 µM) (Figure 2D).

### ARP inhibits migratory ability of U251 glioma cells

**Figure 3:**
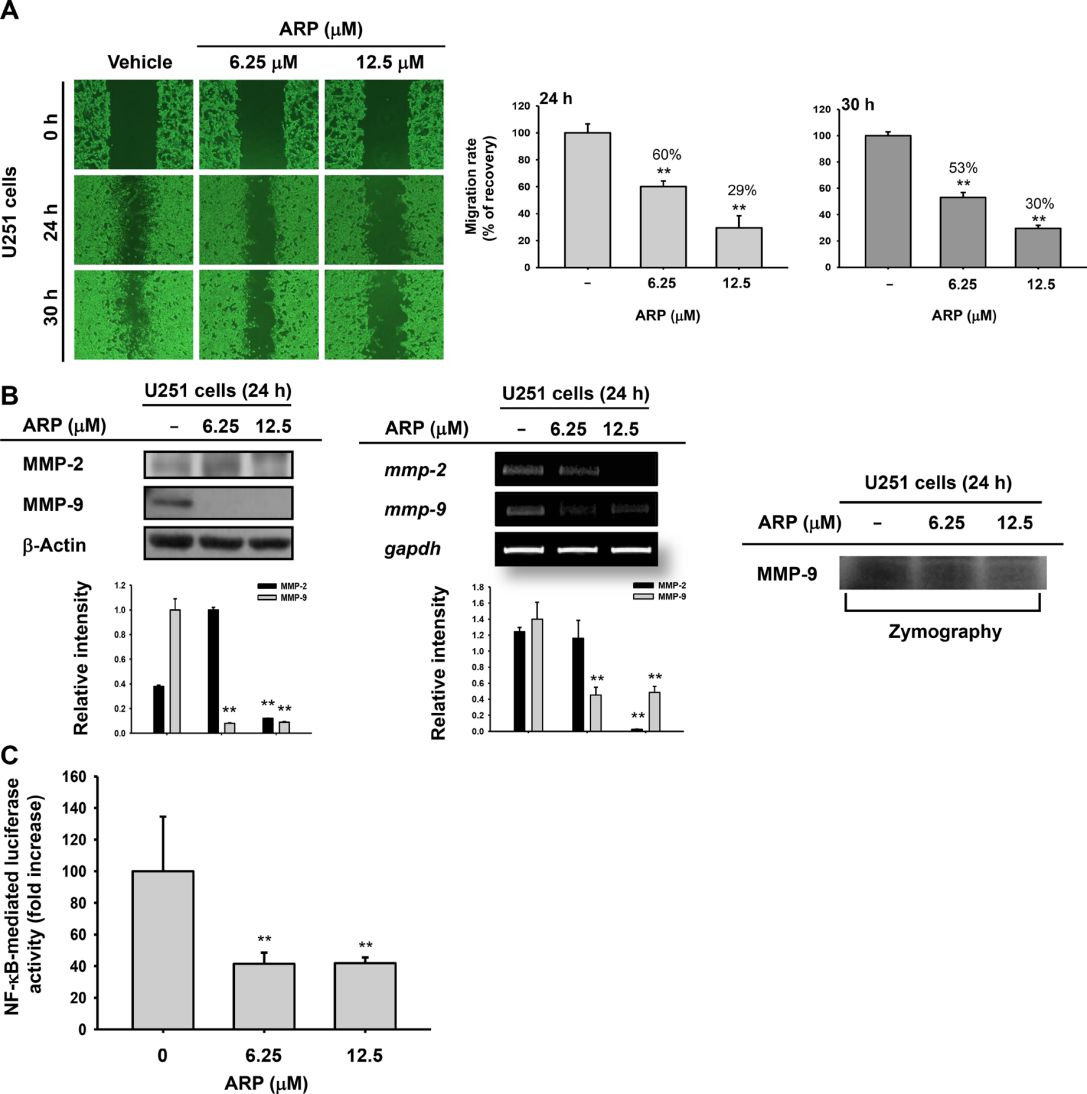
Migration inhibition of U251 glioma cells by ARP (**A**) Migratory ability of U251 glioma cells by wound-healing assays. Wounds were created via scraping with a 200-μl pipette tip, photographed (left), and analyzed (right) 24 and 30 h following ARP treatment. Relative migration rate was evaluated using the DNR Bio-Imaging system. (**B**) Protein (upper), mRNA (middle), and activity (lower) levels of MMP-2 and MMP-9 in ARP-treated U251 cells were analyzed by immunobloting, semi-quantitative PCR, and zymography. (**C**) Promoter-binding activity of NF-κB by luciferase reporter gene assays. U251 glioma cells were transfected with NF-κB-Luc and β-galactosidase for 24 h before ARP treatment. Relative intensity (B) calculated using total MMP-2 and MMP-9 and β-actin or GAPDH by the DNR Bio-Imaging system. ^**^P <0.01 compared with the normal group.

We performed wound-healing assays to evaluate the effects of ARP treatment on U251 glioma cell migration. Cells were treated with nontoxic concentrations of ARP (6.25 and 12.5 µM up to 30 h) to exclude the possibility that reduced migration resulted from cytotoxicity. The migratory ability of U251 cells decreased by 40% at 24 h after treatment with 6.25 µM ARP. This decrease in migratory ability was both time and dose dependent (Figure 3A). A change in *mmp-2* and *mmp-9* expression in migration assays was also examined (Figure 3B). Expectedly, both protein (upper panel of Figure 3B) and mRNA (middle) levels of MMP-2 and MMP-9 as well as the activity of MMP-9 (lower panel) were decreased by ARP at 12.5 µM, according to immunoblotting, RT-PCR, and zymographic analyses (Figure 3B). We also investigated the regulatory effect of ARP on activation of NF-κB using luciferase reporter assay with ARP-treated U251 glioma cells. NF-κB activity was significantly suppressed following ARP treatment (Figure 3C).

### ARP suppresses Src activation

**Figure 4:**
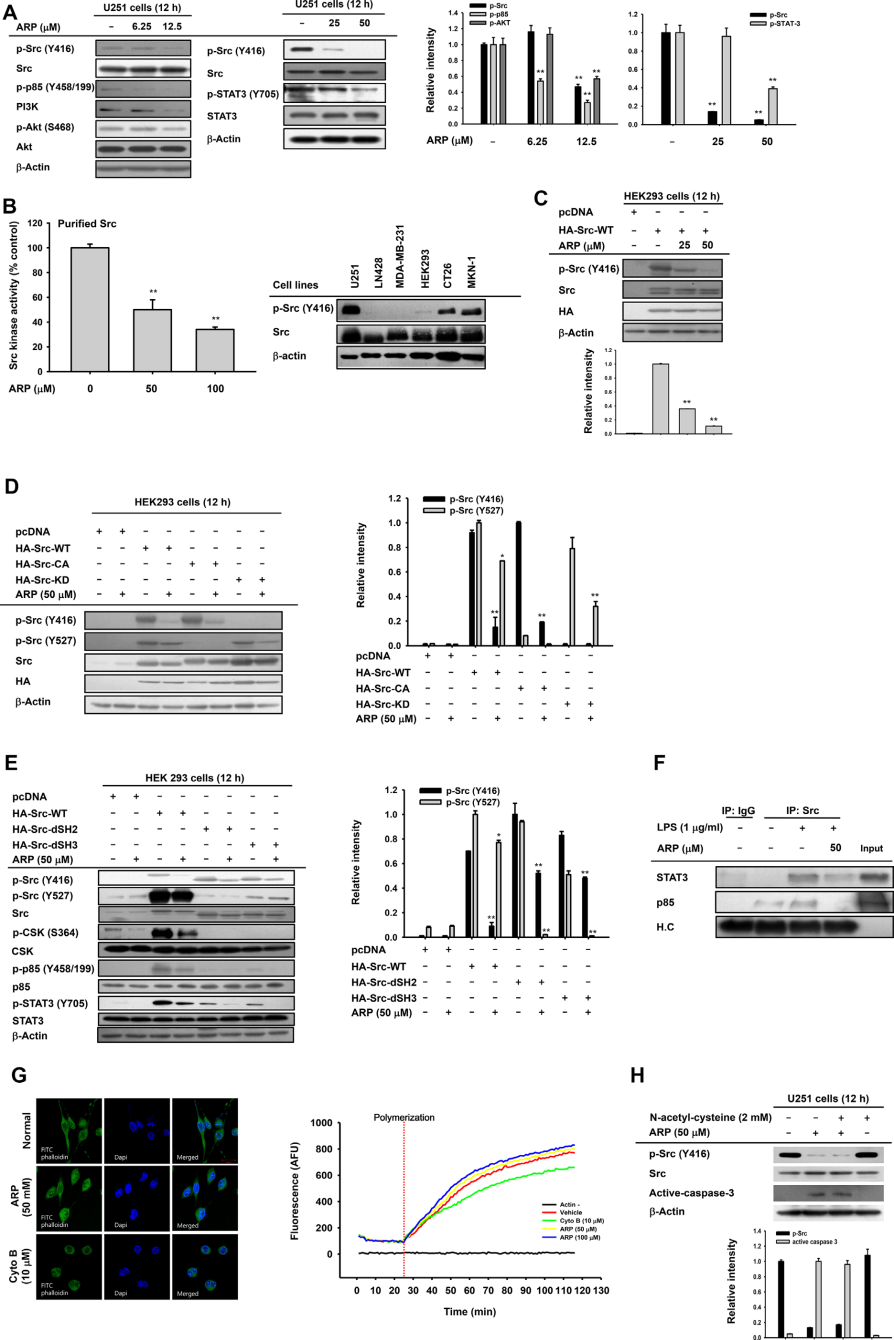
Effect of ARP on Src activation in U251 glioma cells (**A**, left). Decreased phosphorylation of Src, phosphatidylinositide 3-kinase (PI3K), protein kinase B (PKB or Akt), and signal transducer and activator of transcription 3 (STAT3) in ARP-treated U251 glioma cells by immunoblots. Following ARP, activation of migratory proteins (phospho-Src, phospho-PI3K, and phospho-Akt) and survival proteins (phospho-Src and phospho-STAT3) in U251 cells determined by immunoblots. (**B**, left) Src activity by *in vitro* kinase assays used purified Src enzyme. Vehicle control was 100% Src kinase activity. (B left, C, D, and E) Whole cell lysates from U251, LN428, HEK293, CT26, MKN-1, or MDA-MB-231 cells (B, left) or HEK293 cells with ARP following transfection with HA-Src (**C**), constitutively active HA-Src (HA-Src CA), kinase-dead HA-Src (HA-Src-KD) (**D**), or HA-Src SH2 (HA-Src-dSH2) or SH3 (HA-Src-dSH3) domains deleted (**E**). In cell lysates, phosphorylation of Src, CSK, p85, and STAT3 were evaluated by immunoblots. (**F**) Binding of p85 and STAT3 to Src in LPS-treated RAW264.7 cells. (**G**) Effect of ARP on actin polymerization by actin filament-staining with FITC-labeled phalloidin (right) and *in vitro* actin polymerization assays (right). (**H**) Effects of anti-oxidant (N-acetyl-L-cysteine) on suppression of Src phosphorylation and induction of active caspase 3 after ARP in U251 cells by immunoblots. Relative intensity (A right, C, D right, E right, and G) calculated using total p-Src (Y416), p-Src (Y527), p-85/PI3K, active caspase-3, and p-AKT and total forms by DNR Bio-Imaging system. ^*^P < 0.05 and ^**^P < 0.01 compared with the normal or control group.

To understand the anti-cancer mechanism of ARP, a signaling cascade involved in cell proliferation, migration, and metastasis including total and phosphorylated forms of Src, Akt, and PI3K in ARP-treated U251 glioma cells was examined by immunoblotting. Phosphorylation of Src and STAT3 was suppressed by ARP at 12.5 to 50 µM, which was effective in migration assays and apoptosis analysis, in U251 cells (Figure 4A). Since Src is a common enzyme upstream of PI3K and STAT3 [21, 22], Src direct suppression by ARP was examined. *In vitro* Src kinase assays showed suppression by ARP of the enzyme activity of Src, indicating that Src was a target of ARP (Figure 4B left panel). Cell lines U2651, CT26, and MKN-1 with higher IC_50_ values also exhibited higher levels of phospho-Src (Figure 4B right panel). To investigate this possibility, HA-Src and its mutant forms were transfected into HEK293 cells and ARP blocking of Src auto-phosphorylation was investigated. Overexpressing Src resulted in enhanced phospho-Src, while ARP reduced this upregulation (Figure 4C). To further examine how ARP suppressed Src activation, we transfected constitutively active HA-Src CA and kinase-dead HA-Src KD Src and mutant forms with SH2 or SH3 deleted. Phosphorylation of Src on the Y416 residue of this protein triggered by HA-Src or HA-Src-CA was reduced by ARP (Figure 4D). Y416 phosphorylation increased with HA-Src-dSH2 and HA-Src-dSH3 and was weakly attenuated by ARP as in the case of STAT3 phosphorylation (Figure 4E). The level of phospho-Src on Y527 displayed various patterns, depending on the construct. Phosphorylation on Y527 appeared with wild type Src and HA-Src-KD (Figure 4D and 4E). It was weak or not seen after transfection with HA-Src-KD, HA-Src-KD, HA-Src-dSH2, or HA-Src-dSH3 (Figure 4D and 4E). ARP weakly blocked the phosphorylation of Y527 in transfections with wild type and strongly blocked phosphorylation with HA-Src-KD (Figure 4D left and 4E left panels). Similarly, ARP failed to upregulate the activation of CSK, assessed by measuring its phosphorylation level in S364, responsible for increasing the phosphorylation of Y527 (Figure 4E). For direct evidence about whether Src bound its substrates p85 or STAT3, we used immunoprecipitation. Whole cell lysates with increased activated p85 and STAT3 were from LPS-treated RAW264.7 cells, according to previous reports [23]. ARP at 50 µM blocked binding of p85 to Src. STAT3 was partially bound in Src complexes under ARP treatment (Figure 4F).

Since we found that Src inhibition was linked to the suppression of the actin cytoskeleton, we examined whether ARP is able to directly modulate actin polymerization, as adenosine dialdehyde does. Unlike cytochalasin B (10 µM), ARP did not affect actin polymerization at the cellular levels (Figure 4G, left panel) or the protein (G-actin) levels (right panel). In addition, abrogative effect of anti-oxidant N-acetyl-L-cysteine on downregulated phospho-Src and increased active caspase 3 in U251 cells during ARP exposure was not observed (Figure 4H), indicating that reactive oxygen species (ROS) might not be involved in ARP-mediated anti-cancer activity.

### PP2 induces apoptosis and inhibits migratory ability of U251 glioma cells

**Figure 5:**
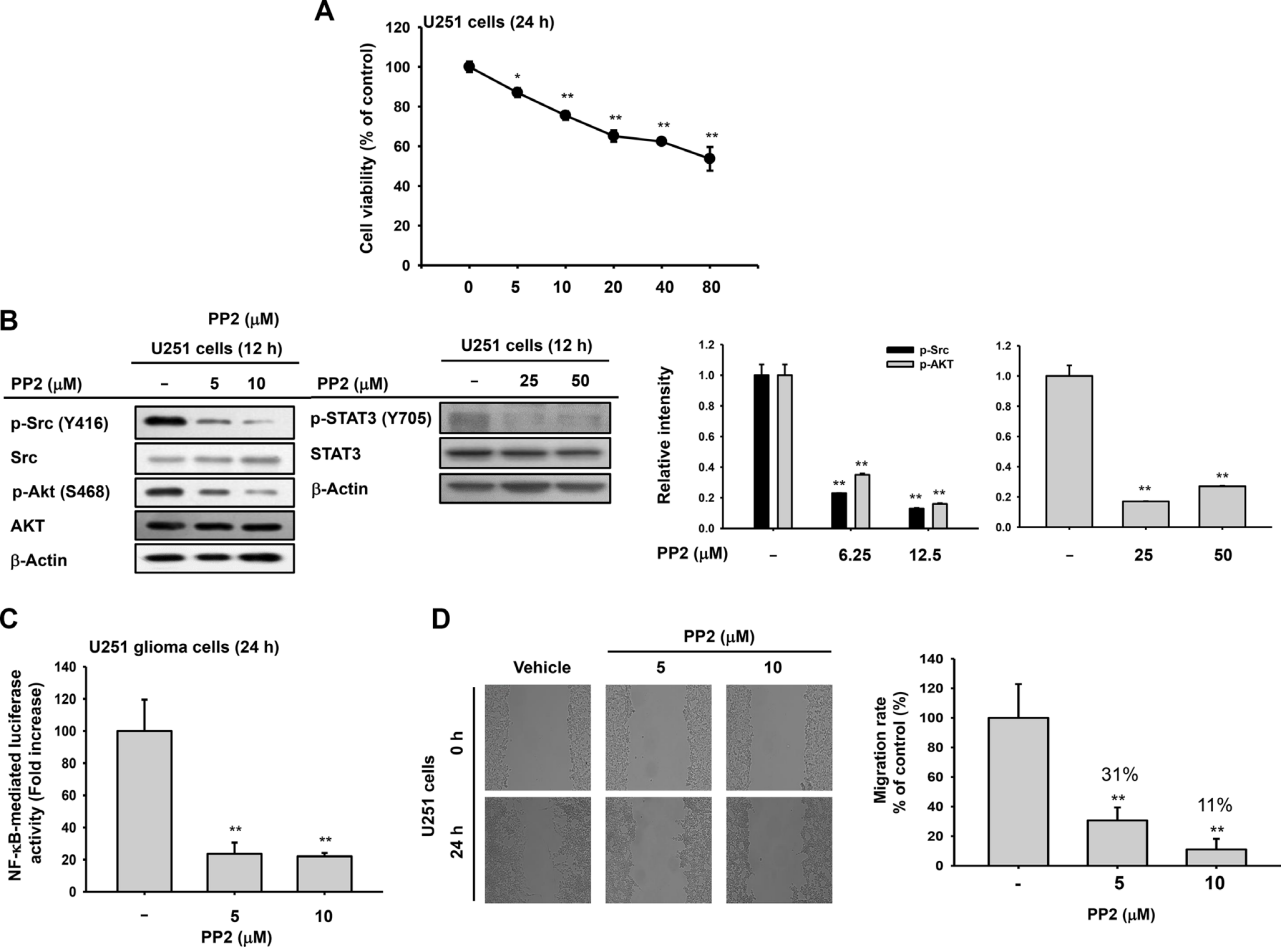
Antitumor effects of Src inhibitor PP2 on U251 glioma cells (**A**) Cytotoxic effects of PP2 by MTT assays 24 h after treatment. (**B**) Decreased phosphorylation of Src, Akt, and STAT3 in PP2-treated U251 glioma cells by immunoblots. (**C**) Decrease in NF-κB activity in U251 glioma cells after PP2 by luciferase reporter gene assays. U251 glioma cells were transfected with NF-κB-Luc and β-galactosidase for 24 h before ARP treatment. (**D**) Suppressed migration of U251 glioma cells following PP2 treatment determined by wound-healing assays. ^*^P < 0.05 and ^**^P < 0.01 compared with the normal group. Relative intensity (B right) calculated using total p-Src, p-AKT, and p-STAT-3 and total forms by DNR Bio-Imaging system. ^**^P < 0.01 compared with the normal group.

To investigate if ARP-suppressive Src was linked to pro-apoptosis and anti-migrative conditions in cancer cells, the Src inhibitor PP2 was employed [24]. PP2 induced cytotoxic effects (Figure 5A) and reduced phosphorylation of Akt, STAT3, and Src (Figure 5B). It suppressed NF-κB activity (Figure 5C) and decreased the migratory ability (Figure 5D) of U251 glioma cells in a dose-dependent manner.

## DISCUSSION

We found that ARP reduced the viability of a number of cancer cell types including glioma cells (Figure 1B, 1C, 1D, and 1E). Cancer cells can be killed by apoptosis or necrosis. As necrosis induces inflammation in healthy cells surrounding a target, it is not considered an ideal mechanism for anticancer drugs [25]. Using several apoptotic markers [26, 27], we confirmed that the cytotoxic activity of ARP resulted from apoptosis. Apoptotic bodies and chromatin condensation were observed in ARP-treated U251 glioma cells, supporting the hypothesis that ARP induced apoptosis in cancer cells (Figure 2). The percentage of late apoptotic cells following ARP treatment also dose-dependently increased (Figure 2B). Additionally, active caspases, which regulate apoptosis [10, 28], increased in ARP-treated U251 cells (Figure 2C left panel). Simultaneously, it was found that treatment of Z-VAD-FMK, a pan caspase inhibitor, reduced ARP-induced cytotoxicity (Figure 2C right panel), implying that activated caspases 9/3 could play a major role in pro-apoptotic and anti-proliferative activities of ARP. In particular, the apoptosis inhibitor Bcl-2 decreased at the transcriptional and translational levels with ARP (Figure 2C and 2D).

We also examined the effect of ARP treatment on U251 glioma cell migration. ARP treatment regulated migration of U251 glioma cells in a dose-dependent manner (Figure 3A), suggesting that ARP may be involved in glioma cell metastasis [29]. The mRNA expression and protein levels of MMP-2 and MMP-9, critical factors in cancer cell migration [30], as well as the activity of MMP-9 also decreased or increased with ARP (Figure 3B). ARP also suppressed NF-κB promoter activity (Figure 3C). Regulatory proteins involved in migratory and apoptotic activities are closely linked. For example, phosphorylation of STAT3 affects Bcl-2 and p53 in parallel, and the complex of NF-κB and phosphorylated Akt is linked to the MMP-9 promoter [31]. Regardless of the cytotoxic activity of ARP, U251 glioma cell migration can be inhibited by reducing Akt/PI3K signaling, which regulates NF-κB activation [31].

Oncogenic Src kinase is important for survival, proliferation, migration, and invasion of cancer cells [2, 4, 7]. As a result, many anticancer drugs target Src directly (e.g., dasatinib for glioma treatment) [5, 6]. The activity of these agents against solid tumors suggests they may also be useful for treating brain metastases [32]. As Src is upstream of PI3K and STAT3 [21, 22], we investigated whether ARP targeted Src. We found that ARP treatment suppressed phosphorylation (Figure 4A), auto-phosphorylation (Figure 4C), and kinase activity (Figure 4B) of Src, implying that ARP might directly suppress Src kinase activity. In addition, cell lines U251, CT26, and MKN-1 cells had higher levels of p-Src and higher anticancer sensitivity toward ARP based on lower IC_50_ values (Figure 4B right panel and Table 1). Under ARP-treated conditions, the lack of inhibition of actin polymerization, an upstream event that activates Src activity in cancer and immune cells (Figure 4F) [33, 34], also indicates that actin was not targeted by ARP in its Src inhibitory action. Based on that N-acetyl-L-cysteine failed to abrogate both Src inhibition and caspase 3 activation by ARP (Figure 4G), we could assume that ROS was not a direct mediator of ARP-induced anti-cancer activity. We obtained similar anticancer activity with PP2, a common Src inhibitor, regarding cell proliferation, inhibition of AKT and STAT3 pathways, and cell migration when it was treated to U251 glioma cells (Figure 5). These results strongly suggested that ARP also inhibited Src kinase and provided a rationale for further investigating Src inhibitors for glioma treatment. Several kinds of Src-modifying plasmids, a constitutively active form (HA-Src-CA) and a kinase-dead form (HA-Src-KD), and forms with SH2 (HA-Src-dSH2) or SH3 (HA-Src-dSH3) domains deleted, were transfected into HEK293 cells to study the effect of ARP treatment on Src (Figure 4D and 4E). Src kinase is activated upon the phosphorylation of tyrosine 416 (Y416) but not Y527 [35]. ARP treatment suppressed phosphorylation of both residues (Y416: a site normally phosphorylated by autophosphorylation, and Y527: a site phosphorylated by CSK) when wild type Src (Src-WT) was transfected (Figure 4D left and Figure 4E left panels). When Src mutants without SH2 and SH3 domains (Src-dSH2 and Src-dSH3 constructs) were transfected, ARP treatment did not strongly reduce Src phosphorylation at Y416 compared to Src-WT-transfected group (Figure 4E panel). In particular, the facts that 1) Y527 phosphorylation in Src-wild type (Src-WT) was not enhanced by ARP, and 2) the phosphorylation of Y527 in the kinase-dead (Src-KD) form was still decreased by ARP treatment indicated that suppression of Y416 phosphorylation but not Y527 phosphorylation could be targeted by ARP. In agreement with these results, CSK activation, which is important for Src activity, was rather diminished by ARP (Figure 4E left panel). These findings seem to imply that SH2/SH3 domains might be important structural units in ARP-mediated inhibition of Src activity derived by suppression of Y416 phosphorylation. Since no clear binding site for ARP was defined in these domains, further detailed experiments are needed with preparation of more mutants in the Src SH2 or SH3 domains.

Gliomas are tumors in the brain or spine that account for approximately 30% of all brain and central nervous system tumors and 80% of all malignant brain tumors [36]. Gliomas vary in aggressiveness and malignancy, but are generally aggressive and infiltrative and have a poor prognosis [32]. A glioma stem cell (GSC) subpopulation has been identified in glioblastoma and is likely a key to the resistance of these tumors to conventional therapies and to recurrent disease [37]. GSCs upregulate a number of signaling pathways required for maintaining neural stem cell stemness. This feature enables them to enhance stemness and aberrant cell survival, leading to tumorigenesis [38, 39]. The Src-linked STAT3/MMP pathway is known to be required for GSC maintenance, partially through upregulating the expression of Toll-like receptor 9 (TLR9) [40, 41]. Stimulation of TLR9 with a CpG ligand (CpG oligodeoxynucleotide) activated STAT3 pathway signaling and promoted GSC growth, whereas silencing TLR9 expression abrogated GSC development [42]. Phosphorylation of STAT3 by Src is also strongly linked to apoptosis [43]. Therefore, ARP-dependent inhibition of Src/STAT3 pathway seems to support the potential of ARP for brain tumor treatment (Figure 4A).

**Figure 6:**
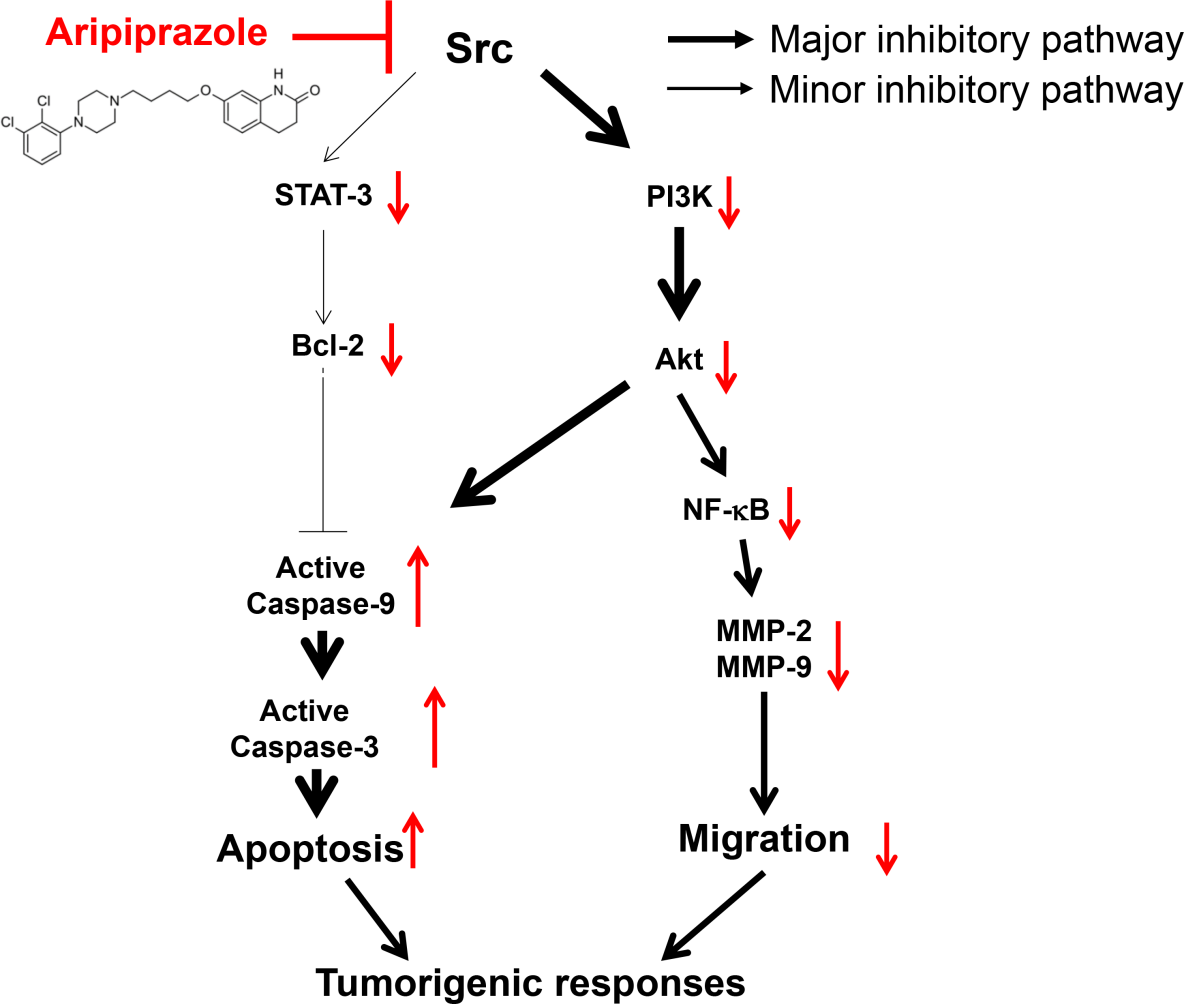
Schematic of the antitumorigenic mechanisms of ARP in U251 glioma cells

As summarized in Figure 6, ARP induced apoptosis and suppressed the migration of glioma cells by targeting Src in *in vitro* and *in vivo* anti-cancer activities. Currently, several anticancer drugs on the market or under development inhibit Src kinase. One example is dasatinib, produced by Bristol-Myers Squibb and sold under the trade name Sprycel. It is used for patients with chronic myelogenous leukemia as a Src family tyrosine kinase and Bcr-Abl tyrosine kinase inhibitor [44]. ARP can also inhibit cancer stem cells and reverse chemoresistance [45]. Our findings suggest that ARP may be useful as an anticancer drug that directly inhibits Src kinase, especially for glioma. Because ARP is being used clinically, development of ARP as Src-targeted anti-cancer drug would be advantageous through drug repositioning that would reduce the cost and time for development of a new drug [46].

## MATERIALS AND METHODS

### Cell lines and cell culture conditions

The human glioma cell lines U251 and LN428, the human gastric adenosquamous carcinoma cancer cell line MKN-1, the human breast cancer cell line MDA-MB-231, the mouse colon carcinoma cell line CT26, and the human embryonic kidney cell line HEK293 were from the American Type Culture Collection (Manassas, VA, USA). U251, MDA-MB-231, CT26, and HEK293 cells were cultured in Dulbecco’s modified Eagle’s medium (DMEM) containing 10% heat-inactivated fetal bovine serum (FBS) (Thermo Fisher Scientific, Waltham, MA, USA). MKN-1, RAW264.7, and LN428 cells were maintained in Roswell Park Memorial Institute (RPMI) 1640 medium (Thermo Fisher Scientific) supplemented with 10% FBS. All cell lines were cultured at 37°C in 5% CO_2_. For all experiments, cells were harvested using a trypsin/ethylenediaminetetraacetic acid solution and were used at 5 to 15 passages.

### Mice

Male Balb/c mice (6–8 weeks old, 17–21 g) were maintained in mouse cages under standard conditions. Water and food (Samyang, Daejeon, Korea) were supplied *ad libitum*. Studies were performed according to the guidelines established by the Institutional Animal Care and Use Committee at Sungkyunkwan University, Suwon, Korea.

### Antibodies, DNA constructs, and reagents

Phospho-specific and total antibodies against caspase-3 and -9; B-cell lymphoma 2 (Bcl-2); matrix metalloproteinase 2 (MMP-2); MMP-9; Src; signal transducer and activator of transcription 3 (STAT3); p85/phosphatidylinositide 3-kinase (PI3K); protein kinase B (PKB or Akt); C-terminal Src kinase (CSK); human influenza hemagglutinin (HA); and β-actin were from Cell Signaling Technology (Beverly, MA, USA). ARP (purity > 98%), N-acetyl-L-cysteine, lipopolysaccharide (LPS), cytochalasin B, 3-(4, 5-dimethylthiazol-2-yl)-2,5-diphenyltetrazolium bromide (MTT), dimethyl sulfoxide, Z-VAD-FMK, and the kinase inhibitor PP2 were from Sigma (St. Louis, MO, USA). Fluorescein isothiocyanate (FITC) Annexin V Apoptosis Detection Kits I were from BD Biosciences (San Diego, CA, USA). HA-Src wild type (HA-Src-WT) and its mutant forms [HA-Src-CA (constitutive-active form), HA-Src-KD (kinase-dead form), HA-Src-dSH2 (SH2-deleted form), and HA-Src-dSH3 (SH3-deleted form) were used as reported previously [34, 47, 48].

### Cell proliferation assays

After 5 × 10^5^ cells/ml of testing cells (in 100 µl) were plated in 96-well plates and pre-incubated for 18 h, ARP (in 100 µl) was added to the culture media at specified doses for an additional 24 or 48 h. The effect of ARP on cytotoxicity was evaluated with conventional MTT assays [49]. Cells were incubated for 3–4 h with MTT solution (10 µl/well) and reactions stopped with the addition of 15% sodium dodecyl sulfate to solubilize formazan [10].

### *In vivo* tumorigenic responses by xenograft mouse model

A xenograft mouse model was generated as reported previously [50, 51]. Briefly, CT26 cells (10^4^ cells in 0.1 ml medium) were injected subcutaneously using a 27-gauge needle. The length and width of tumors were measured using a digital caliper, and tumor volume was determined using the formula: volume = length (largest measurement)/2 x (width)^2^. After anesthetizing with urethane, mice were sacrificed to remove tumor tissues.

### Morphological changes and confocal microscopy

To observe morphological changes, U251 cells were plated in 12-well plates and treated with indicated concentrations of ARP. Images were obtained with an inverted phase contrast microscope attached to a video camera and captured by National Institutes of Health (NIH) imaging software ImageJ. For confocal microscopy, U251 cells were plated on sterile cover slips in 12-well plates and treated with 25 or 50 µM ARP. After 24 h, cells were washed with phosphate-buffered saline (PBS), fixed with 3.7% formaldehyde, and washed three times with PBS. Blocking was with 1% bovine serum albumin (BSA) in PBS, and cells were treated with Hoechst dying solution (1:1000) for nuclear staining. Coverslips were washed with PBS and mounted on glass slides using fluorescent mounting medium (DakoCytomation, Carpentaria, CA, USA). For cytoskeleton staining, FITC-phalloidin (Molecular Probes, 1:250) was added in 1% BSA and incubated for 1 h in the dark. Coverslips were washed three times with PBS. Alexa 488-conjugated secondary antibody (1:100) in 1% BSA was added and incubated for 1 h with shaking at room temperature. Coverslips were washed three times with PBS and mounted onto slides using fluorescent mounting medium (DakoCytomation, Carpentaria, CA). Intensity changes in DAPI and the cytoskeleton were imaged with an Olympus LX70 FV300 (Olympus, Tokyo, Japan).

### Annexin V-propidium iodide staining

Apoptotic cells were visualized based on changes in phosphatidylserine location from inside to outside the cell membrane using FITC-Annexin V Apoptosis Detection Kits [52]. U251 cells were plated in 12-well plates at 5 × 10^5^ cells/mL and treated with ARP at indicated doses. After 24 h, cells were harvested with trypsin, washed with PBS, and resuspended in 1× binding buffer. Cells were incubated with annexin V and propidium iodide (PI) for 15 min at room temperature in the dark. Fluorescence was detected using a BD FACScan flow cytometer and CellQuest Pro (IVD) software (Becton Dickinson, Mountain View, CA, USA).

### Preparation of cell lysates, immunoblotting and immunoprecipitation

Whole cell lysates from ARP-treated U251 and HEK293 cells were analyzed by immunoblotting as reported previously [53]. Levels of total, active, and phosphorylated proteins (caspase 3, caspase 9, Bcl-2, MMP-2, MMP-9, Src, p85, Akt, STAT3, CSK, HA, and β-actin) were visualized with an enhanced chemiluminescence system [54]. For immunoprecipitation, whole cell lysates (500 µg/sample) from RAW264.7 cells (1 × 10^7^ cells/ml) treated or untreated with LPS (1 mg/ml) for 2.5 min were pre-cleared with 10 ml protein A-coupled Sepharose beads (50% v/v) (Amersham, UK) for 1 h at 4°C. Precleared samples were incubated with 5 µl antibody to Src overnight at 4°C. Immune complexes were mixed with 10 µl protein A-coupled Sepharose beads (50% vv) and rotated for 3 h at 4°C.

### mRNA analysis using semi-quantitative and real-time polymerase chain reactions

Total RNA was isolated from ARP-treated U251 cells using TRIzol reagent (Thermo Fisher Scientific) according to the manufacturer’s instructions [55]. Semi-quantitative and real-time polymerase chain reaction (PCR) analyses were conducted as previously reported [56]. All primers (Bioneer, Daejeon, Korea) to analyze the expression levels of *bcl-2, mmp-2, mmp-9,* and *gapdh* are listed in Table 2.

**Table 2:** List of PCR primers used in this study

Name		Sequence (5′ to 3′)
Real-time PCR		
Bcl-2	F	GAAACCCCTAGTGCCATCAA
	R	GGGACGTCAGGTCACTGAAT
GAPDH	F	GGAAGGTGAAGGTCGGAGTCA
	R	GTCATTGATGGCAACAATATCCACT
RT-PCR		
Bcl-2	F	TGTGGCCTTCTTTGAGTTCG
	R	TCACTTGTGGCTCAGATAGG
MMP-2	F	CCCACTGAGGAGTCCAACAT
	R	CATTTACACGTCGGATCT
MMP-9	F	TCCCTGGAGACCTGAGAACC
	R	GGCAAGTCTTCCGAGTAGTTT
GAPDH	F	GCACCGTCAAGGCTGAGAAC
	R	ATGGTGGTGAAGACGCCAGT

### Wound healing assays

U251 cells were plated in 12-well plates at 1.5 × 10^6^ cells/ml, and wounds were generated with a 200-ml pipette tip. Cells were treated with ARP or the kinase inhibitor PP2 at indicated doses. Cell migration was observed with an inverted phase contrast microscope attached to a video camera and captured using NIH imaging software [11].

### Gelatin zymography

The activity of MMP-9 secreted in the conditioned medium was measured by zymography, as reported previously [34]. Gels were stained with Coomassie brilliant blue and destained in methanol/acetic acid (30/10%, v/v).

### Plasmid transfection and luciferase reporter gene assays

HEK293 cells were plated in 12- or 24-well plates. Cells in 12-well plates were transfected with 1.6 mg/ml plasmid containing β-galactosidase and HA-Src, and cells in 24-well plates were transfected with 0.8 mg/ml plasmid containing β-galactosidase and nuclear factor-κB (NF-κB)-Luc for 24 h with the polyethyleneimine method [57]. Cells were treated with ARP or PP2 at indicated concentrations for 12 or 24 h. Luciferase activities were evaluated with the Luciferase Assay System (Promega, Madison, WI, USA).

### *In vitro* kinase assays

The effect of ARP on the activity of purified Src kinase *in vitro* was explored using a kinase profiler service from Millipore (Billerica, MA, USA), as reported previously [58].

### *In vitro* actin polymerization assays

The effect of ARP on actin polymerization was examined according to the manufacturer’s protocol of Actin Polymerization Biochem Kits™ (Cat. No.: BK003, Cytoskeleton, Inc., Denver, CO, USA).

### Statistical analysis

All data presented are expressed as mean ± standard deviation (SD) of experiments performed with six (Figures 1B, 1C, 1D, 1E, 3C, 5A, and 5C) or three (Figures 2C, 2D left panel, 2D right panel, 3A, 3B, 4A, 4B, 4C, 4D, 4E, 4F, 4G, 5A, and 5D) samples for *in vitro* experiments and seven mice for *in vivo* tests (Figure 1F). For statistical comparisons, results were analyzed using ANOVA/Scheffe’s post hoc test or Kruskal-Wallis/Mann-Whitney test. A *P*-value < 0.05 was considered a statistically significant difference. All statistical tests were carried out using the computer program SPSS (SPSS Inc., Chicago, IL). Similar experimental data were observed using an additional independent set of *in vitro* and *in vivo* experiments was conducted using the same numbers of samples or mice.
